# Altered SPECT ^123^I-iomazenil Binding in the Cingulate Cortex of Children with Anorexia Nervosa

**DOI:** 10.3389/fpsyt.2016.00016

**Published:** 2016-02-16

**Authors:** Shinichiro Nagamitsu, Rieko Sakurai, Michiko Matsuoka, Hiromi Chiba, Shuichi Ozono, Hitoshi Tanigawa, Yushiro Yamashita, Hayato Kaida, Masatoshi Ishibashi, Tatsuki Kakuma, Paul E. Croarkin, Toyojiro Matsuishi

**Affiliations:** ^1^Department of Pediatrics and Child Health, Kurume University School of Medicine, Fukuoka, Japan; ^2^Graduate School of Medicine, Kurume University, Fukuoka, Japan; ^3^Department of Psychiatry, Kurume University School of Medicine, Fukuoka, Japan; ^4^Center of Diaginostic Imaging, Kurume University Hospital, Fukuoka, Japan; ^5^Department of Radiology, Kinki University Faculty of Medicine, Osakasayama, Japan; ^6^Department of Radiology, Kurume University School of Medicine, Fukuoka, Japan; ^7^Biostatistics Center, Kurume University School of Medicine, Fukuoka, Japan; ^8^Department of Psychiatry and Psychology, Mayo Clinic, Rochester, MN, USA

**Keywords:** anorexia nervosa, cingulate cortex, GABA, children, iomazenil SPECT

## Abstract

Several lines of evidence suggest that anxiety plays a key role in the development and maintenance of anorexia nervosa (AN) in children. The purpose of this study was to examine cortical GABA(A)-benzodiazepine receptor binding before and after treatment in children beginning intensive AN treatment. Brain single-photon emission computed tomography (SPECT) measurements using ^123^I-iomazenil, which binds to GABA(A)-benzodiazepine receptors, was performed in 26 participants with AN who were enrolled in a multimodal treatment program. Sixteen of the 26 participants underwent a repeat SPECT scan immediately before discharge at conclusion of the intensive treatment program. Eating behavior and mood disturbances were assessed using Eating Attitudes Test with 26 items (EAT-26) and the short form of the Profile of Mood States (POMS). Clinical outcome scores were evaluated after a 1-year period. We examined association between relative iomazenil-binding activity in cortical regions of interest and psychometric profiles and determined which psychometric profiles show interaction effects with brain regions. Further, we determined if binding activity could predict clinical outcome and treatment changes. Higher EAT-26 scores were significantly associated with lower iomazenil-binding activity in the anterior and posterior cingulate cortex. Higher POMS subscale scores were significantly associated with lower iomazenil-binding activity in the left frontal, parietal cortex, and posterior cingulate cortex (PCC). “Depression–Dejection” and “Confusion” POMS subscale scores, and total POMS score showed interaction effects with brain regions in iomazenil-binding activity. Decreased binding in the anterior cingulate cortex and left parietal cortex was associated with poor clinical outcomes. Relative binding increases throughout the PCC and occipital gyrus were observed after weight gain in children with AN. These findings suggest that cortical GABAergic receptor binding is altered in children with AN. This may be a state-related change, which could be used to monitor and guide the treatment of eating disorders.

## Introduction

Anorexia nervosa (AN) typically presents in females during adolescence. It is a serious psychiatric illness conferring substantial morbidity and mortality, which manifests as disturbances in eating habits, excessive preoccupation with weight, restricted caloric intake, and body image distortion (1). Although some research regarding the outcome of childhood AN is encouraging in terms of mortality and recovery from AN (2), long-term comorbid psychiatric disorders, such as anxiety disorders and affective disorders, represent unfavorable prognostic factors (3). Anxiety is present in the majority of children with AN prior to abnormal eating or body image distortions (4). Anxiety in children with AN is also associated with decreased body mass index (BMI) (5, 6). Moreover, trait anxiety scales in children show significant positive correlations with eating disorder psychopathology such as “drive for thinness,” “body dissatisfaction,” and “perfectionism” (5).

Several lines of evidence implicate gamma-aminobutyric acid (GABA)ergic neurotransmission in the pathophysiology of anxiety (7). Recently, a large-scale candidate gene study found that allele frequency differences in the GABA receptor SNP, *GABRG1*, are related to levels of trait anxiety in AN and bulimia nervosa (8). Furthermore, elevated GABA(A) receptor levels in the amygdala were reported in activity-based anorexia (ABA), an animal model of the behavioral phenotype of AN (9). Upregulated GABA(A) receptor function may be associated with anxiety in ABA animals. Several neuroimaging studies have shown negative correlations between GABA-benzodiazepine receptor binding activity and severity of anxiety symptoms in adults with panic or traumatic disorders (10, 11). However, to date, no study has examined GABA(A) receptor binding or function in AN. It is possible that GABAergic neurons may play an important role in both premorbid anxiety of AN and the pathogenesis of childhood AN.

Single-photon emission computed tomography (SPECT) is a nuclear medicine tomographic modality employing gamma rays, and in which, injected radionuclides are attached to ligands selective for specific receptors of interest. ^123^I-iomazenil is a radioactive ligand for central-type benzodiazepine receptors, which form a complex with GABA(A) receptors. Thus, ^123^I-iomazenil SPECT measures GABA(A) receptor binding and indirectly assays GABA(A) receptor function. ^123^I-iomazenil is a frequently used radionuclide tracer for presurgical evaluation of patients with refractory partial epilepsy (12). Moreover, recent neuroimaging studies have explored the role of GABAergic inhibitory function in psychiatric disorders, such as schizophrenia, Alzheimer’s disease, and developmental disorders, as well as anxiety disorders including panic and traumatic stress disorders (10, 11, 13–17). In these reports, significant correlations between GABAergic function and dimensional scales measuring anxiety, panic, negative cognitions, and psychiatric status were found.

The aims of this study were to (1) determine if GABA(A) receptor binding is associated with AN symptoms and anxiety in children initiating clinical treatment for AN; (2) determine which brain regions are involved; (3) determine if measures of GABA(A) receptor binding can predict a participant’s clinical outcome; and (4) determine if these measures change with successful treatment. We hypothesized that lower cortical iomazenil-binding activity is associated with greater baseline symptom severity and poor clinical outcome in children with AN.

## Materials and Methods

### Participants

**Figure 1:**
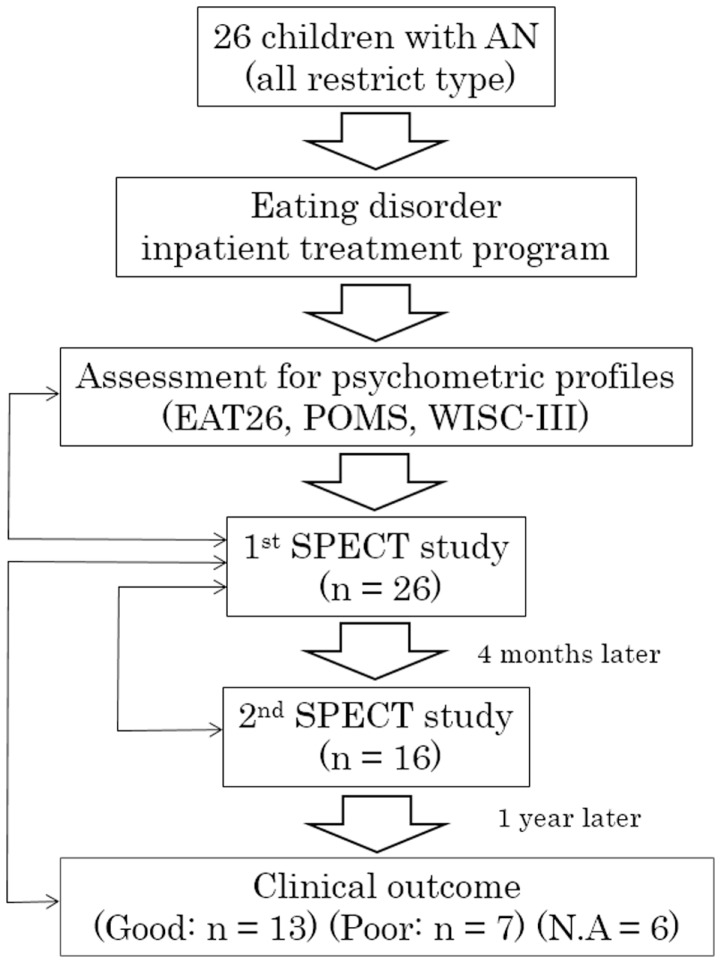
**Flow diagram of the study**. AN, anorexia nervosa; EAT26, Eating Attitude Test with 26 items; POMS, Profile of Mood States; WISC-III, Wechsler Intelligence Scale for Children; SPECT, single-photon emission computed tomography; N.A., not available. Bidirectional thin arrows indicate statistical comparisons or correlation analyses between objects.

The study complies with the Declaration of Helsinki and informed consent was obtained from participants and parents or legal guardians prior to enrollment in the imaging study. The procedures for assent, informed consent, and study design were approved by the Medical Ethical Committee of Kurume University School of Medicine. Twenty-six female participants were recruited who fulfilled the Diagnostic and Statistical Manual of Mental Disorders, 4th Edition (DSM-IV) criteria for AN, and had been admitted to the Department of Pediatrics, Kurume University School of Medicine between 2007 and 2012 for clinical treatment in an eating disorders program. All were restricted-type AN. The flow diagram for the study is shown in Figure 1. The eating disorder treatment program has a multimodal approach, which includes parenteral nutrition, psychotherapy, and behavioral intervention. On initiation of treatment, participants and families have extensive psychoeducation focused on major physical risk factors associated with restricted body weight and therapeutic goals for hospitalization. Individual behavior therapy with reward reinforcement is used to facilitate recovery. Although oral feeding was sufficient for the majority of participants, parenteral nutrition was implemented for select participants with severe AN. Behavioral therapy was combined with nutritional counseling and individual psychotherapy to target difficult emotions and family relational stress. As part of the treatment, participants completed the Eating Attitude Test (EAT-26), a standardized, self-report measure of eating disorder symptoms, which is widely used for screening and measurement of symptoms and characteristics of eating disorders (18). Participants rated their mood using the short form of the Profile of Mood States (POMS), a validated measure that consists of 30 items describing six moods: “Tension–Anxiety,” “Depression–Dejection,” “Anger–Hostility,” “Vigor,” “Fatigue’, and “Confusion” (19). High Vigor scores reflect a good mood or emotion, and low scores in the other subscales reflect a good mood or emotion. Total mood disturbance (TMD) was obtained by subtraction of the Vigor score from the sum of Tension–Anxiety, Depression–Dejection, Anger–Hostility, Fatigue, and Confusion scores. Each original POMS score was converted to a *T*-score (20). We selected the POMS for measurement of anxiety, as we have neither a Japanese version of State-Trait Anxiety Inventory for Children (STAIC) nor other validated Japanese psychometric scales for anxiety. Upon enrollment in the study, a diagnosis of AN and comorbidities was confirmed in all participants by semi-structured interviews using the Mini-International Neuropsychiatric Interview (MINI) (21), which were performed by two psychiatrists (Michiko Matsuoka and Hiromi Chiba). All participants underwent cognitive assessment using the Wechsler Intelligence Scale for Children (WISC-III). Psychometric profiles were performed before treatment. Participants were medication naïve and did not receive pharmacological treatment during the course of the study. Twenty-three participants had secondary amenorrhea and three had not yet reached menarche. In all participants, brain magnetic resonance imaging (MRI) examination was performed on admission to identify any structural abnormalities, e.g., regional brain atrophy. Participants with severe, co-occurring medical illnesses (such as superior mesenteric artery syndrome) were excluded. Ethical concern regarding the use of ionizing radiation in healthy children precluded the enrollment of a control group for this study.

### Clinical Outcome Measures

Follow-up clinical assessments were performed 1 year after hospital discharge by one pediatrician (Shinichiro Nagamitsu) and two psychiatrists (Michiko Matsuoka and Hiromi Chiba). A structured approach was used to define clinical outcome *a priori*. Clinical outcome score was based on eight items, as defined in prior work (22). This included weight change, menstrual status, abnormal eating behavior, body image, binge eating or purging behaviors, insight, school attendance, and quality of family relationship. Improved or impaired answers were scored “0” and “1,” respectively. For items of weight change and school attendance, improved and unimproved ratings were scored “0” and “2,” respectively. The middle score “1” indicates “unchanged condition.” The outcome was considered good with total scores less than “4” and poor with total scores of “4” or over. The clinical outcome raters (Shinichiro Nagamitsu, Michiko Matsuoka, and Hiromi Chiba) were blinded regarding SPECT data.

### Iomazenil SPECT

All 26 children underwent brain imaging using SPECT. The first ^123^I-iomazenil SPECT examination was performed before treatment and the second one immediately before discharge (16 of 26 participants). Mean duration between the first and second SPECT examinations was approximately 4 months. Briefly, participants were injected intravenously with a bolus of 95–117 MBq ^123^I-iomazenil (Nihon Medi-Physics Co., Tokyo, Japan), which binds with high affinity to the GABA(A)-benzodiazepine receptor. The SPECT scan was performed 3 h after injection of the tracer without any sedation, using a large field-of-view dual-detector camera and a computer system equipped with a low-energy, high-resolution, and parallel-hole collimator. The dual detector camera rotated over 180° in a circular orbit and in 32 steps of 40 s each to cover 360° in approximately 22 min.

### Image and Statistical Analyses

**Figure 2:**
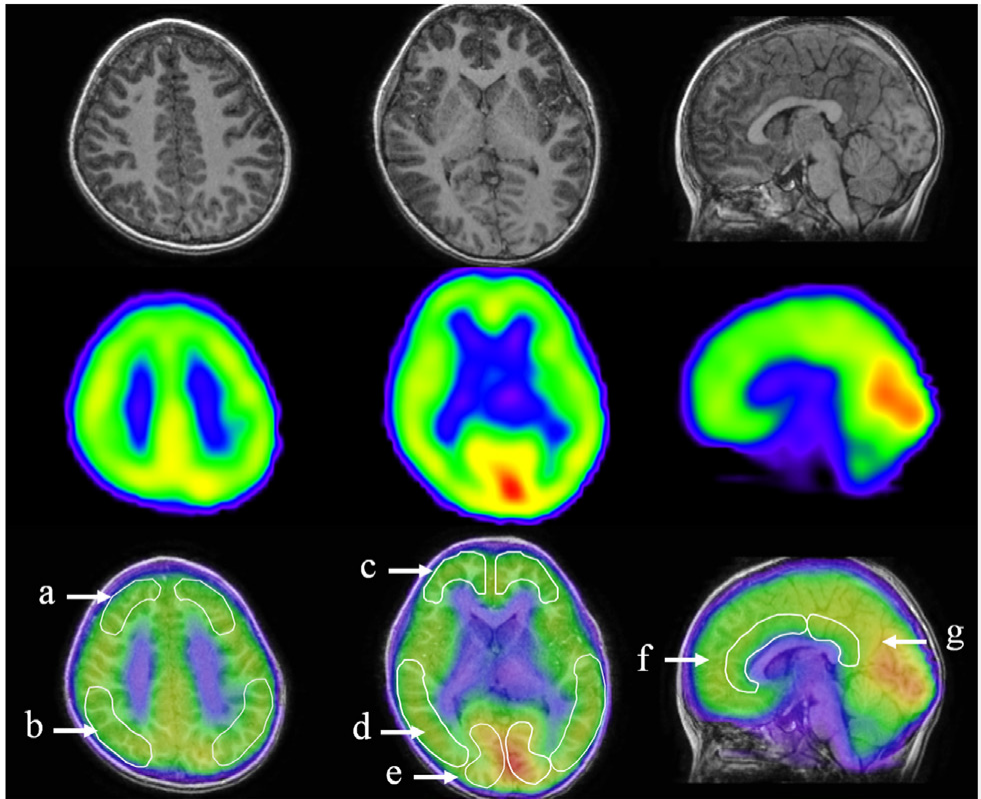
**Designated regions of interest (ROIs) in fusion images of ^123^I-iomazenil SPECT and MRI**. The top panel shows brain MRI (transverse and sagittal T_1_ sequences), the middle panel the corresponding results of ^123^I-iomazenil SPECT, and the bottom panel fusion imaging. Outlined regions in the bottom panel indicate designated ROIs, namely, **(a)** the superior frontal, **(b)** parietal, **(c)** frontal, **(d)** middle temporal, and **(e)** occipital regions; **(f)** anterior and **(g)** posterior cingulate gyrus.

Images of ^123^I-iomazenil scintigraphs were analyzed by three-dimensional stereotactic surface projections (3D-SSP) using iSSP3 software (Nihon Medi-Physics Co.). Stereotactic anatomical standardization was performed as described previously (23). Briefly, rotational correction of the SPECT data set and three-dimensional centering were performed, followed by realignment to the anterior commissure–posterior commissure line. Differences in individual brain size were accounted for by linear scaling and regional anatomical differences minimized using a non-linear warping technique (24). Consequently, each brain was anatomically standardized to match a standard atlas brain. Brain MRI was performed using a superconducting magnet operating at 1.5 T. For coregistered SPECT and MRI analysis, a method of image integration was applied using Fusion Viewer software (Nihon Medi-Physics Co.) with a registration algorithm based on maximum mutual information (Figure 2). Subsequently, cortical and subcortical regions of interest (ROIs) in the acquired SPECT data were defined. Using elliptical templates, ROIs were drawn manually for the major cortical and subcortical brain regions in a representative subject. To eliminate the disadvantage of lower reliability with manual operations, the same elliptical templates were used to define ROIs in other subjects. ROIs were placed over the following regions: superior frontal, middle frontal, parietal, middle temporal, and occipital regions; the cerebellum in each hemisphere; and the anterior and posterior cingulate cortex (ACC and PCC, respectively; Figure 2). Two neuroradiologists (Hitoshi Tanigawa and Masatoshi Ishibashi), blinded to clinical symptoms, independently drew ROIs. Each relative iomazenil-binding activity in ROIs was expressed as a ratio of that in the cerebellum, as patients with AN have no cerebellar symptoms. Spearman’s correlation was used to determine correlations between relative iomazenil binding in each region on baseline SPECT scan and age, BMI-standard deviation score (BMI-SDS), EAT-26, and POMS subscale score. To test for possible differential relationships between POMS and iomazenil-binding activity in brain regions, the brain regions were classified into three groups: center region, left hemisphere region, and right hemisphere region. ROIs were grouped accordingly: ACC and PCC as the center region; left of superior frontal, parietal, frontal, temporal, and occipital as the left hemisphere region; and right of superior frontal, parietal, frontal, temporal, and occipital as the right hemisphere region. POMS subscales were separately analyzed using the mixed-effect model (SAS 9.3 PROC MIXED). Regions, POMS subscale, and their interactions were treated as fixed effects in the model, while the intercept was treated as a random effect, therefore accounting for correlations among iomazenil-binding activities. When the interaction between all three regions and POMS subscale was significant, ROIs were analyzed within the region and POMS using mixed model regression to determine significances between ROIs. The Mann–Whitney *U* test was used to compare between participants with good and poor outcomes.

Neurological Statistical Image Analysis software (NEUROSTAT, Stat_1tZ), which can perform a paired *t* test between two corresponding groups using cross-sectional images, was adopted to examine changes in iomazenil binding between the first and second SPECT. Statistical significance was set at *Z*-score >2, a level commonly used to discriminate abnormalities. The regions identified were transformed into three-dimensional anatomical data and Talairach coordinates to show brain landmarks.

## Results

### Participants’ Characteristics

The mean and SD of age before treatment was 14.1 (1.3) years of age (range, 10.5–15.6 years of age) (Table 1). Mean (SD) BMI before and after treatment were 13.7 (2.0) and 15.7 (0.9), respectively. Mean (SD) BMI-SDS before and after treatment were −3.7 (1.9) and −2.3 (1.1), respectively. Mean (SD) EAT-26 score before therapy was 22.4 (12.0), which was higher compared with the reference control value (25). Five participants had co-occurring psychiatric disorders including two with autism spectrum disorders, one with learning disability, and two with selective mutism. Mean (SD) scores for each of the POMS subscales were 46.1 (10.3) for Tension–Anxiety, 53.1 (11.8) for Depression–Dejection, 50.3 (11.9) for Anger–Hostility, 43.4 (11.8) for Vigor, 47.4 (11.4) for Fatigue, and 52.1 (15.5) for Confusion. Mean (SD) score for TMD of POMS was 205.6 (59.4). The subscale scores of Tension–Anxiety and Depression–Dejection were higher than normal ranges (26), but the differences did not reach significance. Mean (SD) IQ was 105 (13). None of the participants showed regional brain atrophy on brain MRI examination. Mean duration of hospitalization was approximately 4 months. Mean (SD) age on the second SPECT examination was 14.8 (1.1). The range of duration between the first and second SPECT was from 86 to 250 days (mean, 128 days).

**Table 1:** **Clinical characteristics and POMS scores for subjects with relative iomazenil-binding activity in each brain region**.

	Before treatment (1st SPCET)		After treatment (2nd SPCET)
	All subjects	Subjects having 2nd SPECT	
*N*	26	16	16
Age	14.1 ± 1.3	14.4 ± 1.1	14.8 ± 1.1
BMI	13.7 ± 2.0	13.3 ± 1.6	15.7 ± 0.9
BMI-SDS	−3.7 ± 1.9	−4.1 ± 1.9	−2.3 ± 1.1
EAT-26	22.4 ± 12.0		
Menstrual cycle			
Not experienced	2		
Secondary amenorrhea	22		
POMS			
Tension–Anxiety	46.1 ± 10.3		
Depression–Dejection	53.1 ± 11.8		
Anger–Hostility	50.3 ± 11.9		
Vigor	43.4 ± 11.8		
Fatigue	47.4 ± 11.4		
Confusion	52.1 ± 15.5		
Total score	205.6 ± 59.4		
WISC-III	105 ± 13		
Relative iomazenil-binding activities			
R superior frontal	1.41 ± 0.11	1.40 ± 0.11	1.45 ± 0.12
L superior frontal	1.44 ± 0.15	1.45 ± 0.13	1.48 ± 0.16
R parietal	1.46 ± 0.14	1.48 ± 0.14	1.50 ± 0.14
L parietal	1.51 ± 0.16	1.55 ± 0.15	1.53 ± 0.13
R middle frontal	1.41 ± 0.11	1.41 ± 0.11	1.46 ± 0.10
L middle frontal	1.45 ± 0.14	1.47 ± 0.14	1.52 ± 0.12
R middle temporal	1.46 ± 0.12	1.46 ± 0.12	1.54 ± 0.12*
L middle temporal	1.44 ± 0.14	1.47 ± 0.14	1.54 ± 0.11*,^†^
R occipital	1.74 ± 0.14	1.78 ± 0.14	1.88 ± 0.26*
L occipital	1.77 ± 0.20	1.79 ± 0.19	1.88 ± 0.21^‡^
Anterior cingulate	1.44 ± 0.13	1.47 ± 0.12	1.59 ± 0.24*,^†^
Posterior cingulate	1.65 ± 0.17	1.69 ± 0.16	1.80 ± 0.33^‡^

*SPECT, single-photon emission computed tomography; BMI, body mass index; EAT-26, Eating Attitude Test with 26 items; POMS, Profile of Mood States; WISC-III, Wechsler Intelligence Scale for Children; R, right; L, left*.

*Significant difference compared with all subjects (*P* < 0.05).

*^†^Significant difference compared with subjects at second SPECT (*P* < 0.05)*.

*^‡^Trend toward significance in all subjects (*P* < 0.1)*.

### Participants’ Outcome

Clinical outcome scores 1 year after treatment were examined in 20 out of 26 participants. Three participants did not complete the treatment. It was not possible to examine three other participants, as two were transferred to locked psychiatric units and one was transferred to a local hospital. After the evaluations, 13 participants were classified as having a good outcome and 7 with a poor outcome.

### Baseline Correlations between ^123^I-iomazenil Binding and Clinical Measures

Relative iomazenil-binding activities in each brain region are summarized in Table 1. There were significant associations between some clinical measures and relative iomazenil-binding activity in several brain regions. Higher EAT-26 scores were significantly associated with lower iomazenil-binding activity in the ACC (Table 2). Higher “Tension–Anxiety” score at the beginning of therapy was significantly associated with lower iomazenil-binding activity in the left superior frontal, parietal, middle frontal cortex, and PCC (Table 2). Higher “Anger–Hostility,” “Confusion,” and “Total” scores at the beginning of therapy were significantly associated with lower iomazenil-binding activity in the same regions and left occipital cortex (Table 2). Furthermore, “Depression–Dejection” and “Fatigue” scores were also significantly associated with lower iomazenil-binding activity in the PCC (Table 2). There were no associations between BMI-SDS and iomazenil-binding activity in any brain region. However, there were significant positive correlations between age and iomazenil-binding activity in the left and right middle frontal, left parietal, and PCC (*r* = 0.415, 0.392, 0.430, and 0.454, respectively, *P* < 0.05) (data not shown).

**Table 2:** **Correlation coefficients between POMS subscale scores, EAT-26, and iomazenil-binding activity in each brain region from the first SPECT**.

	TA	D	AH	V	F	C	Total	EAT-26
R superior frontal	0.036	0.290	−0.034	−0.014	0.161	0.097	0.116	−0.203
L superior frontal	−0.473*	−0.428*	−0.494*	0.155	−0.332	−0.646**	−0.530*	−0.338
R parietal	0.022	0.312	−0.037	−0.057	0.019	−0.001	0.073	−0.100
L parietal	−0.413*	−0.250	−0.414*	−0.029	−0.314	−0.604**	−0.417*	−0.338
R frontal	−0.213	0.057	−0.179	−0.073	0.054	−0.094	−0.061	−0.431
L frontal	−0.498*	−0.320	−0.468*	0.100	−0.288	−0.598**	−0.476*	−0.390
R temporal	−0.095	0.226	−0.241	−0.144	0.015	−0.009	0.009	−0.151
L temporal	−0.210	−0.130	−0.367	−0.040	−0.101	−0.374	−0.245	−0.141
R occipital	−0.175	0.003	−0.356	−0.111	−0.162	−0.222	−0.168	−0.109
L occipital	−0.0355	−0.378	−0.510*	0.027	−0.288	−0.589**	−0.454*	−0.097
Anterior cingulate	−0.043	−0.008	−0.034	−0.277	−0.016	−0.156	−0.005	−0.606**
Posterior cingulate	−0.553*	−0497*	−0.476*	0.262	−0.440*	−0.641**	−0.594*	−0.312

*POMS, Profile of Mood States; TA, Tension–Anxiety; D, Depression–Dejection; AH, Anger–Hostility; V, Vigor; F, Fatigue; C, Confusion; EAT-26, Eating Attitude Test with 26 items*.

*P < 0.05 and **P < 0.01 indicate significant correlations.

### Interactions between POMS Subscales and Brain Regions in Iomazenil-Binding Activity

The mixed-effect model detected significant interaction effects between three main brain regions and POMS total scale and subscales of “Depression–Dejection” and “Confusion” (Table 3). In the three main brain regions, significant differences were identified between the left and right hemisphere regions on POMS total score and subscales of “Depression–Dejection” and “Confusion” (Table 4). Furthermore, significant differences between left and right hemisphere regions were identified in the superior frontal region on POMS total score (*t* = −2.63, *P* = 0.0094), superior frontal and occipital regions on the subscale of “Confusion” (*t* = −3.16, *P* = 0.0018; *t* = −3.49, *P* = 0.0062, respectively), and superior frontal region on the subscale of “Depression–Dejection” (*t* = −2.75, *P* = 0.0065). This finding remained significant after Bonferroni correction for multiple comparisons (*P* = 0.01).

**Table 3:** **Interaction effects between POMS subscales and three main regions (center, left, and right hemispheres)**.

POMS subscales	df (between regions)	df (within regions)	*F*	*P*-value
POMS total	2	194	3.52	<0.05*
Tension–Anxiety	2	194	2.07	0.13
Depression–Dejection	2	194	4.09	<0.05*
Anger–Hostility	2	194	2.16	0.12
Vigor	2	194	0.22	0.8
Fatigue	2	193	1.49	0.23
Confusion	2	194	5.65	<0.01*

*POMS, Profile of Mood States*.

*Indicates significance.

**Table 4:** **Interaction between three regions and POMS subscales**.

	POMS total		Confusion		Depression–dejection	
	*t*	*P*-value	*t*	*P*-value	*t*	*P*-value
Center–left	0.88	0.38	−1.22	0.22	−0.5	0.62
Center–right	−1.12	0.27	−1.32	0.19	−1.61	0.11
Left–right	2.65	0.0087*	3.36	<0.001*	2.79	0.0057*

*indicates significance.

### Comparison of ^123^I-iomazenil-Binding Activity Before and After Treatment

**Figure 3:**
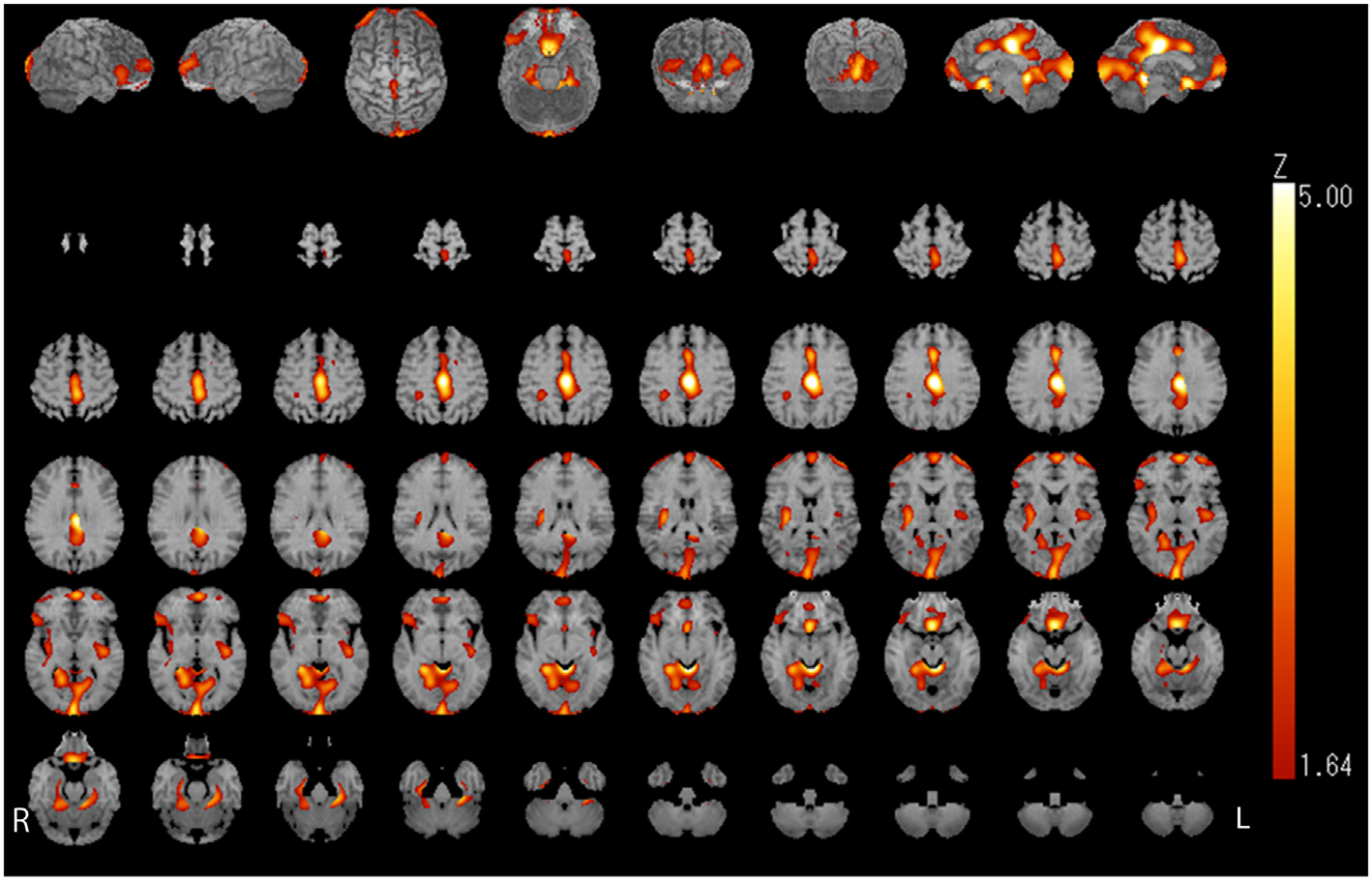
**Image analysis (1tZ) of increased iomazenil-binding changes before and after weight gain in the brain of children with AN**. Significant increases in iomazenil-binding activity before and after weight gain are shown in the anterior and posterior cingulate cortex, occipital cortex, frontal cortex, and hippocampus, as indicated by the bright orange color.

Relative iomazenil-binding activity after treatment was significantly increased in the ACC, right occipital, and bilateral middle temporal gyrus (Table 1). Comparisons of adjusted iomazenil-binding activity before and after weight gain were examined in the same 16 participants using NEUROSTAT. There were significant increases in iomazenil-binding activity after treatment in the ACC, PCC, frontal gyrus, occipital gyrus, and hippocampus (Figure 3). By contrast, there was a significant decrease in iomazenil-binding activity after treatment in the bilateral inferior temporal cortex (data not shown). The Talairach coordinates of sites with *Z*-scores >3.0 are listed with the associated brain regions in Table 5.

**Table 5:** **Brain regions and Talairach coordinates showing significantly increased and decreased iomazenil binding in children with anorexia nervosa before and after weight gain**.

Regions	Talairach coordinates				*P*-value
	*X*	*Y*	*Z*	*Z* score	
Increased regions					
Right anterior cingulate gyrus	3	−19	36	6.33	<0.00001
Right occipital gyrus	17	−103	−4	5.43	<0.00001
Left medial frontal gyrus	−1	19	−14	4.89	<0.00001
Left occipital gyrus	−1	−94	2	4.31	<0.00001
Right posterior cingulate gyrus	6	−40	22	3.99	<0.00005
Right parahippocampal gyrus	26	−31	−25	3.84	<0.0001
Right medial frontal gyrus	3	29	4	3.84	<0.0001
Decreased regions					
Right inferior temporal gyrus	55	−24	−9	4.28	<0.00001
Left inferior temporal gyrus	−53	−19	−14	3.05	<0.01

### Association between ^123^I-iomazenil-Binding Activity in the ACC and Clinical Outcome

**Figure 4:**
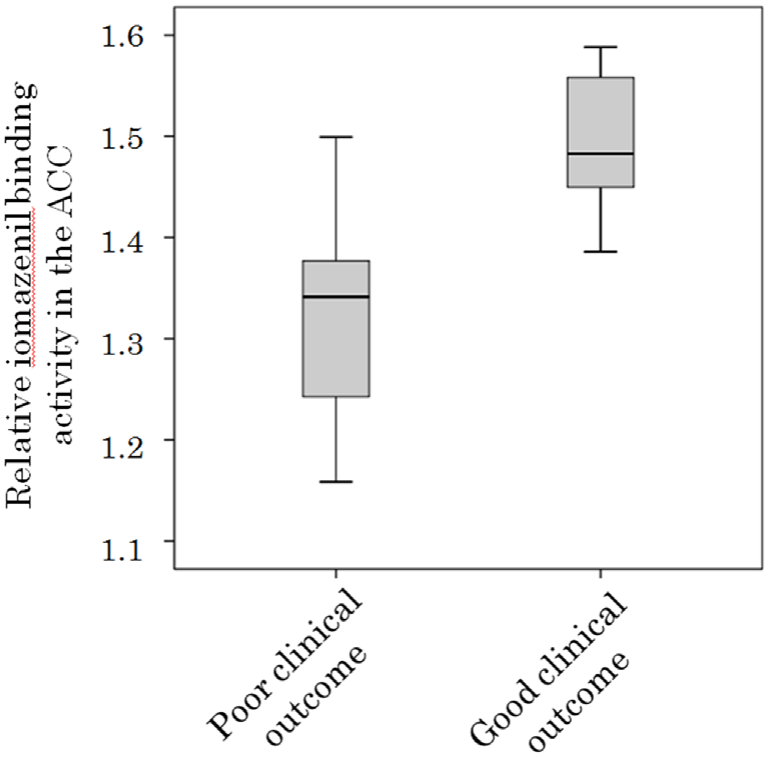
**Relative iomazenil-binding activity in the anterior cingulate gyrus in participants with different clinical outcomes**. Relative iomazenil-binding activity in the anterior cingulate gyrus at initiation of treatment in participants with good clinical outcomes was significantly higher than those with poor clinical outcomes.

There was a significant baseline difference in iomazenil-binding activity between participants with good and poor clinical outcome scores in the ACC (1.48 ± 0.09 vs. 1.32 ± 0.12, respectively, *P* < 0.05) (Figure 4) and left parietal gyrus (1.57 ± 0.12 vs. 1.40 ± 0.15, respectively, *P* < 0.05). Relative baseline iomazenil-binding activity in the ACC and left parietal gyrus in participants with a poor clinical outcome were significantly lower than those with a good clinical outcome.

## Discussion

To our knowledge, this is the first investigation on SPECT ^123^I-iomazenil brain imaging in children with AN. Using SPECT ^123^I-iomazenil brain imaging, we found association between cortical GABAergic receptor binding and clinical manifestations of childhood AN. Higher EAT-26 and mood disturbance scores were significantly associated with lower GABAergic inhibitory binding in various brain regions. Poor clinical outcome was also associated with lower GABAergic receptor binding in the ACC and left parietal region. GABAergic receptor binding was mainly activated in the ACC, PCC, frontal gyrus, and occipital gyrus after treatment.

We found significant correlation between reduced GABAergic receptor binding in various brain regions and mood disturbances, as assessed using POMS subscales. Mixed model regression showed significant effects for the interactions between brain regions and POMS total scale and subscales of “Depression–Dejection” and “Confusion.” Furthermore, the effect of these POMS profiles showed significantly different binding activities between the left and right hemispheres, especially in the superior frontal region. These results indicate that GABAergic neuronal activity correlates to mood disturbances in children with AN. The potential involvement of GABAergic neurotransmission in the pathophysiology of AN was recently investigated by genetic allele frequency analysis of *GABRG1* in AN patients. This study showed significant correlation between specific allele frequency in this GABA receptor SNP and levels of trait anxiety in the patients (8). Further, in an animal model of AN, elevated GABA(A) receptor expression in the amygdala was associated with increased anxiety (9). It remains unclear whether GABA(A) receptor function is associated with the underlying pathophysiology of childhood AN or a result of long-term starvation. However, a relative strength of our present findings is that at repeat SPECT scan, the participants were not completely weight-restored [mean BMI 15.7 (0.9 SD)], suggesting that changes in brain ^123^I-iomazenil binding may be related to clinical improvement rather than mere weight restoration. Nonetheless, this does suggest that GABAergic receptor-mediated inhibitory function may be associated with mood disturbances in children with AN.

We also found a significant negative correlation between ^123^I-iomazenil-binding activity in the ACC and abnormal eating attitude described by EAT-26 score, with a greater decrease in activity in the ACC of AN children with poor clinical outcomes, compared with those with good clinical outcomes. Furthermore, binding activity in the ACC was significantly increased after treatment. As the present clinical outcome score was composed of current BMI and presence of menstruation, as well as changes of eating behavior and social interaction, GABAergic functional activity in the ACC may be related to biological vulnerability of recovery from AN symptoms. Converging lines of evidence suggest correlations between morphological or functional neural changes and differential clinical outcomes in AN. For example, McCormick et al. (27) reported that although the dorsal ACC gray matter volume is significantly reduced in patients with AN compared with normal controls, greater normalization of the right dorsal ACC volume following weight restoration prospectively predicted sustained remission at 1 year post-hospitalization. Functional MRI studies have shown that increased activation in the dorsal ACC and prefrontal cortex in response to food stimuli differentiates recovered AN patients from chronically ill AN patients (28). Further, subcallosal cingulate deep brain stimulation has recently been applied as a treatment strategy for treatment-refractory AN and associated with improvement in mood, anxiety, affective regulation, and increased BMI (29). Taken together, our findings contribute to emerging evidence that variations in functional activities of the ACC may be predictors of outcomes of AN.

Similar to the ACC, neural activities in the PCC may play important roles in the pathophysiology of AN. The PCC is functionally coupled with other brain regions as a default mode network and involved in self-related aspects of cognitive processing such as self-reference and self-reflection (30). Functional brain imaging suggests that dysfunction in resting-state functional connectivity in regions involved in self-referential processing might be associated with development of AN (31). Further, several lines of evidence show that less activation in the PCC is associated with altered inhibitory processing, which might represent a behavioral characteristic and impairment of emotional processing in AN (32, 33). In the present study, several higher mood disturbance scores were significantly associated with lower GABAergic inhibitory binding, mainly in the PCC. Similarly, the PCC was one of the brain regions in which iomazenil-binding activity increased after treatment. In a previous neuroimaging study in children with AN, increased cerebral blood flow (CBF) was observed in the parietal cortex and PCC after inpatient treatment (34). Taken together, increased GABAergic inhibitory function in the PCC after weight gain in our study might indicate improved self-referential processing and cognitive control, which were missing during their starvation period.

We found evidence of increased ^123^I-iomazenil-binding activity in the occipital cortex after treatment in children with AN. In general, iomazenil-binding activity is strongest in the occipital cortex, indicating that GABA receptors are densely distributed in this area. GABA is involved in interocular suppression in the visual cortex and plays a central role in determining visual cortex selectivity (35). As the brain has a limited capacity, attention allows relevant incoming information to be selectively enhanced while suppressing irrelevant information, the processing for which may be modulated by GABAergic inhibitory function (36, 37). A recent MR spectroscopy study revealed negative correlation between the amount of occipital GABA and cognitive failure in healthy patients, indicating that the inhibitory capacity in sensory areas affects their ability to ignore information that is irrelevant to current behavioral goals (37). In AN patients, cognitive deficits in impaired visuospatial ability, impaired complex visual memory, and impaired selective attention have been reported (38, 39). As GABA plays an important part in stimulus processing and suppression in sensory areas, increased GABAergic inhibitory activation observed in the occipital cortex in AN participants in our study may reflect enhanced suppression of visual information processing, possibly resulting in the improvement of cognitive deficits.

The present study has several limitations that require consideration in future studies. First, brain imaging data from normal healthy children are not available because ethics approval was not feasible for SPECT studies in healthy control children. Therefore, we focused our research on the correlation between GABAergic inhibitory function in brain regions and psychometric profiles, and changes in these functions before and after treatment in AN participants. Thus, we could not determine if the basic GABAergic inhibitory function is upregulated or downregulated, compared to healthy controls. Second, it is possible that changes in iomazenil binding after treatment might be associated with confounding effects, secondary to starvation and restored body weight. However, as the average period for repeat SPECT was short (4 months), weight restoration was not complete. Furthermore, our previous neuroimaging report regarding CBF changes after treatment in AN patients showed no global increase in CBF changes except specific brain regions (bilateral parietal lobe and PCC) (34). Consequently, confounding effects are unlikely to be a cause of our SPECT findings. Third, SPECT exhibits poor resolution around some limbic regions, such as the amygdala and hippocampus, which are important for emotion processing. In these small regions, the obtained radioactivity might differ from the true activity because of a partial volume effect (PVE). The PVE can be defined as underestimation of binding per unit brain volume in small objects or regions because of blurring of radioactivity (spill-out and spill-in) between regions (40, 41). These regions need to be resolved using MR imaging-based correction for PVE. Fourth, we did not examine the participants’ psychometric profiles at the end of hospitalization. Although the majority of participants obtained proper eating habits, attended school, and improved parental relationships by the discharge period, these socio-emotional behaviors were not evaluated using the psychometric profile. To confirm our understanding of altered ^123^I-iomazenil-binding activity due to therapeutic intervention, improvement of socio-emotional difficulties should be consecutively measured.

In conclusion, GABAergic inhibitory receptor function in the brain may play an important role in manifesting clinical symptoms of childhood AN. Lower GABAergic ^123^I-iomazenil binding in specific brain regions at initiation of treatment is associated with clinical severity of mood disturbances and abnormal eating attitude in this sample of participants. Decreased binding in the ACC and left parietal cortex were associated with poor clinical outcomes. Conversely, increased changes in GABAergic receptor binding in the ACC, PCC, and occipital gyrus might be important for the recovery process of childhood AN. Although GABAergic function in the cingulate cortex might be evaluated as a potential predictor of clinical outcome, it will be important to determine the association between function and long-term prognosis. Further research focused on GABAergic receptor-mediated function among participants with eating disorders is warranted.

## Author Contributions

SN participated in the design of this study and compiled the manuscript. SN, MM, HC, SO, and YY saw the patients and obtained informed consent and their agreement to participate in the study. Three radiologists (HT, HK, and MI) were in charge of radioactive measurements and calculations of iomazenil activity using ROIs. RS and TK, statistician, conducted the statistical analyses. PC and TM supervised the preparation of the manuscript.

## Conflict of Interest Statement

The authors declare that the research was conducted in the absence of any commercial or financial relationships that could be construed as a potential conflict of interest. The Reviewer, JK, and handling Editor, GH, declared their shared affiliation, and the handling Editor states that the process nevertheless met the standards of a fair and objective review.
